# Evaluating the Impact of the Prime Time Sister Circles^®^ Intervention on Reducing Depressive Symptoms Among African American Women with Uncontrolled Hypertension

**DOI:** 10.1007/s11606-023-08288-z

**Published:** 2023-07-27

**Authors:** Hossein Zare, Chidinma A. Ibe, Manshu Yang, Gayle Porter, Marilyn Gaston, Nicole Jones, Wehmah Jones, Vivienne Rose, Michele Balamani, Denise L. Woods, Darrell J. Gaskin

**Affiliations:** 1 Department of Health Policy and Management, Johns Hopkins Bloomberg School of Public Health, Baltimore, MD USA; 2 University of Maryland Global Campus (UMGC), Adelphi, USA; 3 Department of Health, Behavior and Society, Johns Hopkins Bloomberg School of Public Health, Baltimore, MD USA; 4 Division of General Internal Medicine, Johns Hopkins School of Medicine, Baltimore, MD USA; 5 Johns Hopkins Center for Health Disparities Solutions, Johns Hopkins Bloomberg School of Public Health, Baltimore, MD USA; 6 Department of Psychology, University of Rhode Island, Kingston, RI USA; 7 The Gaston & Porter Health Improvement Center, Inc, Washington, DC USA; 8 American Institutes for Research, Washington, DC USA; 9 University of Maryland School of Medicine, Baltimore, MD USA; 10 Baraka and Associates, Largo, MD USA

**Keywords:** African American Women, PTSC, CES-D-10, depressive symptoms.

## Abstract

**Background:**

The Prime Time Sister Circles®, a randomized controlled trial (PTSC-RCT), assessed the impact of a community-based peer support program on hypertension management among African American women 40–75 years of age. While the PTSC-RCT was designed to evaluate changes in blood pressure control, subsequent sub-analyses revealed a high proportion of self-reported depressive symptoms in our sample. Accordingly, we conducted an ancillary investigation of the PTSC intervention on depression to ascertain its impact on reduced depressive symptoms in the study population.

**Method:**

Depressive symptoms were measured using an adapted version of the Center for Epidemiologic Studies Depression Scale Revised (CES-D-10). We used unadjusted and adjusted fixed effect models. Data for this study came from the PTSC-RCT. We collected data between 2017 and 2018 in Washington, DC. We used a balanced analytical sample of 172 African American, English-speaking women between 40 to 75 years old with uncontrolled hypertension.

**Intervention:**

The intervention group participated in a 2-h, peer-based support group once a week for 13 weeks. A trained PTSC facilitator facilitated sessions with experts who delivered content on various topics, including psychosocial wellness (e.g., stress, depressive symptoms, anxiety management, and self-esteem), physical health (e.g., hypertension, inflammation, and heart disease), physical activity, and healthy nutrition.

**Results:**

Results from the fixed-effects models indicated that participants in the PTSC program exhibited a greater reduction in CES-D-10 score at three months (Coeff: -1.99, 95% CI: -3.49, -0.49) and at 15 months (Coeff: -2.38, 95% CI: -3.94, -0.83), as compared to those in the control group.

**Conclusions:**

Evidence suggests that the Prime Time Sister Circles® intervention reduced depressive symptoms among African American women with low socioeconomic status and hypertension.

**Trial Registration:**

NCT04371614.

## Background

Approximately 18.5% of adults aged 18 and over have reported experiencing any symptoms of depression in 2019,^1^ with higher prevalence observed among low-income African American (AA) women.^2–6^ Several factors have been proposed as contributors to this phenomenon, including residence in environments with higher poverty,^7^ lower access to healthy foods,^8^ lower access to mental health providers due to lack of health insurance,^9–11^ higher prevalence of comorbidities,^12–14^ and a lower rate of social supports.^15^ The promotion and adherence to cultural beliefs such as the “strong Black woman” trope^16–18^ may limit some AA women’s emotional capacity to employ healthy coping strategies that are supportive of their mental health, including seeking care for psychological issues or availing themselves of support from members of their social networks.^19–21^ AA women are more likely than women of other races to face a unique constellation of stressors that can create and sustain depressive symptoms; these stressors include racism, sexism, poverty, and discrimination. AA women's depressive symptoms often differ from White women's, including increased appetite and weight, irritability and persistent physical symptoms.^19^ The COVID-19 pandemic and its impact on the mental health of African Americans at large confers urgency to this pressing issue.^20^

Older AA women have higher rates of depressive symptoms than their White peers.^21^ The association between depressive symptomology and the occurrence of other health conditions is well established: Germain reports that AA women with high depressive symptoms have higher prevalence of functional impairment.^22^ Other studies have reported the co-occurrence of depression and poor cardiovascular health among AA women. Burroughs et al. (2019) reported that AA women were more likely to have high Cumulative Psychosocial Stress (CPS) scores (172.5 ± 54.9) and experience low ideal cardiovascular health (ICH).^23^

The association between depression and many chronic diseases, including but not limited to hypertension,^24^ obesity,^25^ cardio-metabolic disorder syndrome,^26,27^ and cardiovascular diseases (CVD),^28^ can result in higher mortality rates and lower quality of life in AA women, especially those with lower incomes.^29^ Research has shown that AA women with low-income status and hypertension suffer from depressive symptoms, which implies that this is a particularly vulnerable group.^14^ Yet, despite indication suggesting a strong link between high blood pressure and depression,^30,31^ AA women whose health care entails ongoing monitoring of their blood pressure may not be receiving adequate care for their depression;^32^ indeed, only a third of AA adults experiencing mental illness received services in 2019.^33^

While community-based interventions that address the unique behavioral, cultural, emotional, and social needs of low-income AA may be difficult to access due to comorbidities, scheduling, and various social risk factors,^33^ they are a necessary strategy to combat intransigent disparities observed in this population. The *Oh Happy Day Class* is an example of a culturally adapted intervention for African American adults, which reduced depressive symptoms in participants by 6.6 points from baseline to 3-month follow-up.^34^ The Prime Time Sister Circles® (PTSC) Program is another promising intervention. The PTSC Program incorporates peer support and didactic training that focuses on psychosocial health and well-being to: (1) address the sociocultural risk factors that mid- to late-life AA women with uncontrolled hypertension experience and (2) promote the adoption of primary and secondary prevention strategies to control their blood pressure and engagement in strategies geared toward psychosocial health.^33^

We leveraged a randomized controlled trial evaluating the impact of the PTSC intervention on hypertension control to explore its effect on reducing depressive symptoms among mid-life, low-income AA women with uncontrolled hypertension. This was achieved through a secondary analysis of data collected between 2017 and 2018 as a part of this trial. We report on the impact of the PTSC intervention on participants’ depressive symptoms.

## Methods

### Trial design

This study is a secondary analysis of data collected as part of a larger randomized clinical trial that evaluates the effectiveness of the Prime Time Sister Circle® (PTSC) on a range of health, healthcare utilization, and behavioral outcomes. ^33^ Details of the PTSC-RCT are summarized elsewhere.^27^ In short, the PTSC-RCT’s primary outcome is hypertension control, and it was powered to assess the impact of exposure to the PTSC intervention on hypertension. The study’s secondary outcomes include health knowledge, self-efficacy, behaviors, and psychosocial health. For this analysis, we used data from the PTSC-RCT to study the impact of the PTSC intervention on depressive symptoms reported by our study population, low-income AA women with uncontrolled hypertension receiving care in a Federally Qualified Health Center (FQHC). We hypothesized that PTSC intervention would reduce the CESD-10 score in women who participated in the 13-week training session.

### Participants

Data for this study came from the PTSC-RCT. The PTSC was a community-based intervention program targeting English-speaking AA women who were 40 to 75 years of age, had been diagnosed with uncontrolled hypertension (systolic blood pressure ≥ 140 mm Hg and diastolic blood pressure ≥ 90), resided in Washington, D.C., and were patients at Unity Health. Study participants were randomly assigned to the PTSC intervention or the control group, and both arms maintained their care at Unity. We collected data from 2017 to 2018.

### Interventions

The main goals of the PTSC intervention were to help AA midlife women learn how to manage, lower, and control their high blood pressure; expand their repertoire of stress management techniques; increase their physical activity; and improve their nutrition. Study participants were assigned to cohorts of Sister Circles that met once a week, over a 13-week period, for 2 h at a time. The PTSC intervention curriculum included culturally competent modules designed to help AA women manage their stress, an underlying contributor to depression. To that end, two sessions were devoted to stress and depressive symptom management. They were conducted by midlife AA women with extensive mental health expertise (i.e., psychologists or licensed clinical social workers). Each participant was taught general stress-management techniques such as deep breathing, communication skills, and time management strategies. They also developed a stress-management plan. Sister Circle sessions were held in meeting rooms provided by three churches in Washington, D.C.

### Measures

#### Outcomes

To identify participants with depressive symptoms, we employed the Center for Epidemiologic Studies Depression Scale Revised (CES-D-10), a widely used self-reported measure of depression symptoms. The CES-D-10 comprises ten items (8 positively worded and two negatively worded) and has exhibited strong internal consistency (Cronbach’s α = 0.86) and test–retest reliability (ICC = 0.85) among the general population.^35,36^ Further, the CES-D-10 is a reliable tool for measuring depression, with a value of the reliability coefficient of 0.7 (Cronbach’s alpha) and composite reliability of 0.72; ^37^ as such, the scale is an excellent measure to identify individuals at risk for clinical depression.^38^ Each item uses a 4-point Likert scale, with 0 indicating rarely or none of the time (less than one day per week) and 3 indicating all of the time (5‐7 days per week). Following the CES-D-10 guidelines, we reverse coded items 5 and 8 (two items that were negatively worded) and then calculated the total score by summing up scores of the ten items, resulting in a maximum scale score of 30 and a minimum scale score of 0.^37^

#### Control variables

In all models, we controlled for sociodemographic variables at baseline, including age and marital status, education, health insurance status, and health behavior status (smoking or alcohol consumption). We also controlled for several chronic conditions at baseline through a composite measure showing whether a participant had reported diabetes, high blood cholesterol, heart disease, cancer, stroke, and obesity).

#### Sample size

**Figure 1:**
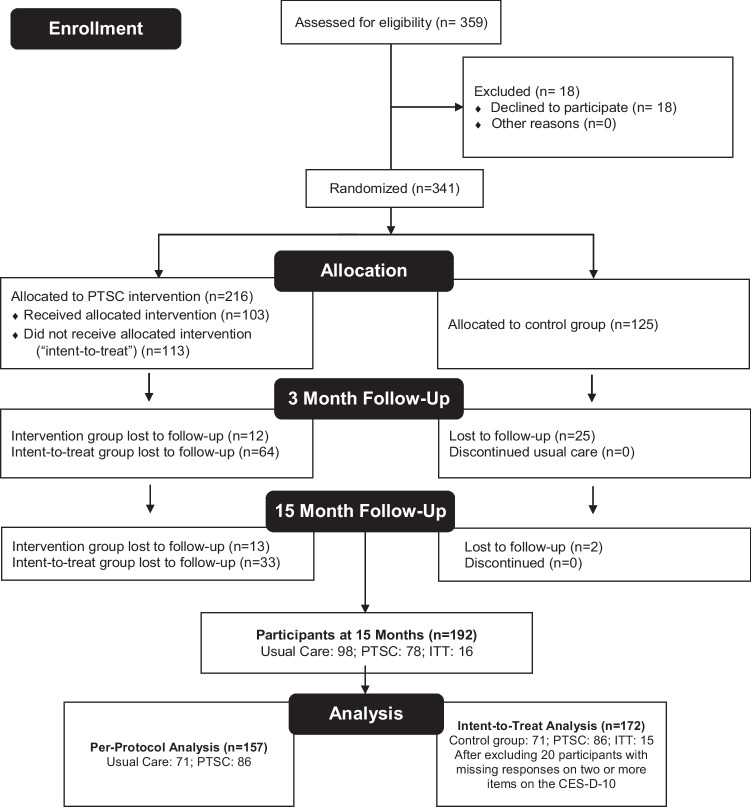
Prime time sister circles randomized clinical trial study at the 3 months and 15 months follow ups (CONSORT Flow Diagram).

We recruited 341 AA women receiving care at a Health Care Center (‘facility’ in this text) in Washington, D.C., in 2017 and 2018. Eligible participants were English-speaking women between 40 to 75 years of age who self-identified as AA, received their primary care at a Unity practice, and had been diagnosed with uncontrolled hypertension (systolic blood pressure ≥ 140 mm Hg or diastolic blood pressure ≥ 90).^2,25^ Participants were randomly assigned to receive the PTSC intervention (*n* = 216) or to the control group and proceeded with their standard clinical care at Unity (*n* = 135). We designated participants randomized to the intervention group, who did not receive it as intended, as the Intent-to-Treat (ITT) group (see CONSORT diagram for more details, Fig. 1). We collected data at baseline, 3 months, and 15 months. Among the 341 participants who provided baseline data, 240 provided data at the 3-month mark, and 217 at the 15-months mark. Notably, nearly 30% of the participants dropped out of the study after randomization. Sub-analyses indicated that social risk factors (e.g., transportation) and personal health concerns were primary drivers for suboptimal participation. For this analysis, we excluded 25 participants with missing responses on two or more items on the CES-D-10. We constrained the analytic sample to those who furnished data during all 3 data collection time points. This reduced the sample to 172 participants (516 observations).

#### Randomization

Details of our randomization strategy have been captured elsewhere.^33^ In summation, recruitment procedures in Washington, DC commenced in 2017 and ended in 2018. The facility generated a list of patients across their practices who met the study's eligibility criteria and shared their names and addresses with the study team. Potential candidate participants received a letter highlighting information about the study, incentives for participation, contact information for study team members, and the dates and locations of the recruitment sessions. Those who were interested were invited to a recruitment meeting to learn about the PTSC-RCT, including specifics about the PTSC. During this meeting, participants were randomly assigned to the PTSC intervention or control groups. Upon providing written consent, study participants completed a baseline survey that was administered verbally by the study team. Data were collected immediately after the intervention (3 months), and one-year post-intervention (15 months).^33^ The CES-D-10 was administered at baseline, three months, and 15 months.

#### Statistical Analysis

We conducted descriptive analyses to compare those in the control group (those receiving usual care), PTSC group (intervention), and Intent-to-Treat (ITT, assigned to PTSC but did not participate in the intervention sessions) groups. Using unequal variance *t*-tests and Chi-Squared tests, we examined potential differences between control, PTSC, and ITT for control variables. Then, we ran a fixed-effect model analysis using a balanced panel, which allowed us to measure the impact of the PTSC program on the same individual 3 months and 15 months after the intervention. We ran sets of unadjusted and adjusted models. For the unadjusted model, we controlled only for the group (PTSC), time (3 months and 15 months), and interaction (being in PTSC/ITT groups at 3 or 15 months as a fixed effect measure to measure the impact of PTSC program at 3 and 15 months). For the adjusted models, we controlled for age, marital status, education, income, health insurance status, smoking status, drinking status, and the number of chronic conditions.

There were some missing values for the control variables, including age, marital status, education, income, health insurance coverage, smoking, and drinking behaviors; we cite the percentage of missing values for these variables in Table 1. To address the issue of missing data, we used the multiple imputations (MI) technique in Stata to fill in the missing values, assuming that data was missing at random. All statistical procedures were conducted using Stata, version 15.

**Table 1:** Distribution of Characteristics Among PTSC-RCT Participants at Baseline for the Balanced Sample, 2017–2018

	Control (*n* = 71)	PTSC (*n* = 86)	ITT (*n* = 15)
	*n* (%)	*n* (%)	*n* (%)
CES-D-10 score, mean (SD)	10.5 (5.7)	9.2 (5.5)	10.6 (6.3)
Age (years), mean (SD)	57.2 (7.6)	59.3(8.1)	57.3(7.3)
Marital Status (%)			
Single without a partner	38 (53.5)	46(53.5)	4(26.7)
Married or Living with a partner﻿	11(15.5)	20(23.3)	6(40.0)
Other (divorced, separated, and widowed participants)﻿	21(29.6)	18(20.9)	5(33.3)
Missing	1(1.4)	2(2.3)	0(0.0)
Education (%)			
Less than high school	15(21.1)	29(33.7)	5(33.3)
High school graduate/GED	36(50.7)	33(38.4)	6(40.0)
More than high school	15(21.1)	18(20.9)	4(26.7)
Missing	5(7.0)	6(7.0)	0(0.0)
Income (%)			
Under $20,000	55(77.5)	65(75.6)	11(73.3)
$20,001-$40,000	11(15.5)	16(18.6)	1(6.7)
$40,001-Above	3(4.2)	3(3.5)	1(6.7)
Missing	2(2.8)	2(2.3)	2(13.3)
Has Health Insurance (%)	70(97.1)	85(98.8)	15(93.3)
Missing	2(1.4)	1(1.2)	0(0.0)
Currently Smoke (%)	33(46.4)	36(42.4)	6(40.0)
Missing	2(2.8)	1(1.2)	0(0.0)
Drink Alcohol (%)	30(41.8)	41(47.6)	9(60.0)
Missing	4(5.6)	4(4.7)	0(0.0)
Number of Chronic Diseases, mean (SD)	2.2(1.4)	2.3(1.3)	2.4(1.5)

GED: general education development, PTSC: Prime Time Sister Circles, ITT: Intent-to-Treat, SD: Standard Deviation. 1) Abbreviations: CES-D, Center for Epidemiologic Studies Depression Scale; GED, general education development. PTSC-RCT, Prime Time Sister Circles randomized control trial. 2) We ran unequal t-tests and chi-sq to test the means between control and PTSC groups and UC and ITT groups. 3) We found no significant differences using unequal variance t-tests and Chi-Squared tests between UC and PTSC, ITT and PTSC groups, and US and ITT

For the Intent-to-Treat analysis, we used balanced panel data for women who participated in all 3 rounds of data collection. We included PTSC and ITT (participants initially assigned to PTSC regardless of whether they received the PTSC treatment) and control. The analytical sample of this analysis was 172 participants (516 observations). We also conducted a Per-Protocol analysis. To do so, we repeated the ITT analysis by excluding those assigned to the intervention group who did not receive the treatment (Intent-to-Treat), which reduced the sample to 140 participants (420 observations). This study was approved by the Institutional Review Boards (IRBs) of the Johns Hopkins Bloomberg School of Public Health (JHBSPH) and the American Institutes for Research (JHBSPH IRB No. 00007121).

## Results

The distribution of characteristics among the PTSC-RCT participants by intervention groups is shown in Table 1. On average, women who participated in the PTSC intervention were 59 years old. They were also more likely to be single without a partner (53.5%), have a high school diploma (38.4%), and have a household income of less than $20,000 a year (75.6%). Our findings showed that more than 42.4% of the PTSC group were current smokers, 47.6% reported some level of alcohol consumption and had been diagnosed with at least 2.3 chronic conditions. In addition, almost all had health insurance coverage (98.8%). There were no significant differences in sociodemographic variables between PTSC, ITT, and control groups except for drinking and smoking. (See Table 1.)

**Fig. 2:**
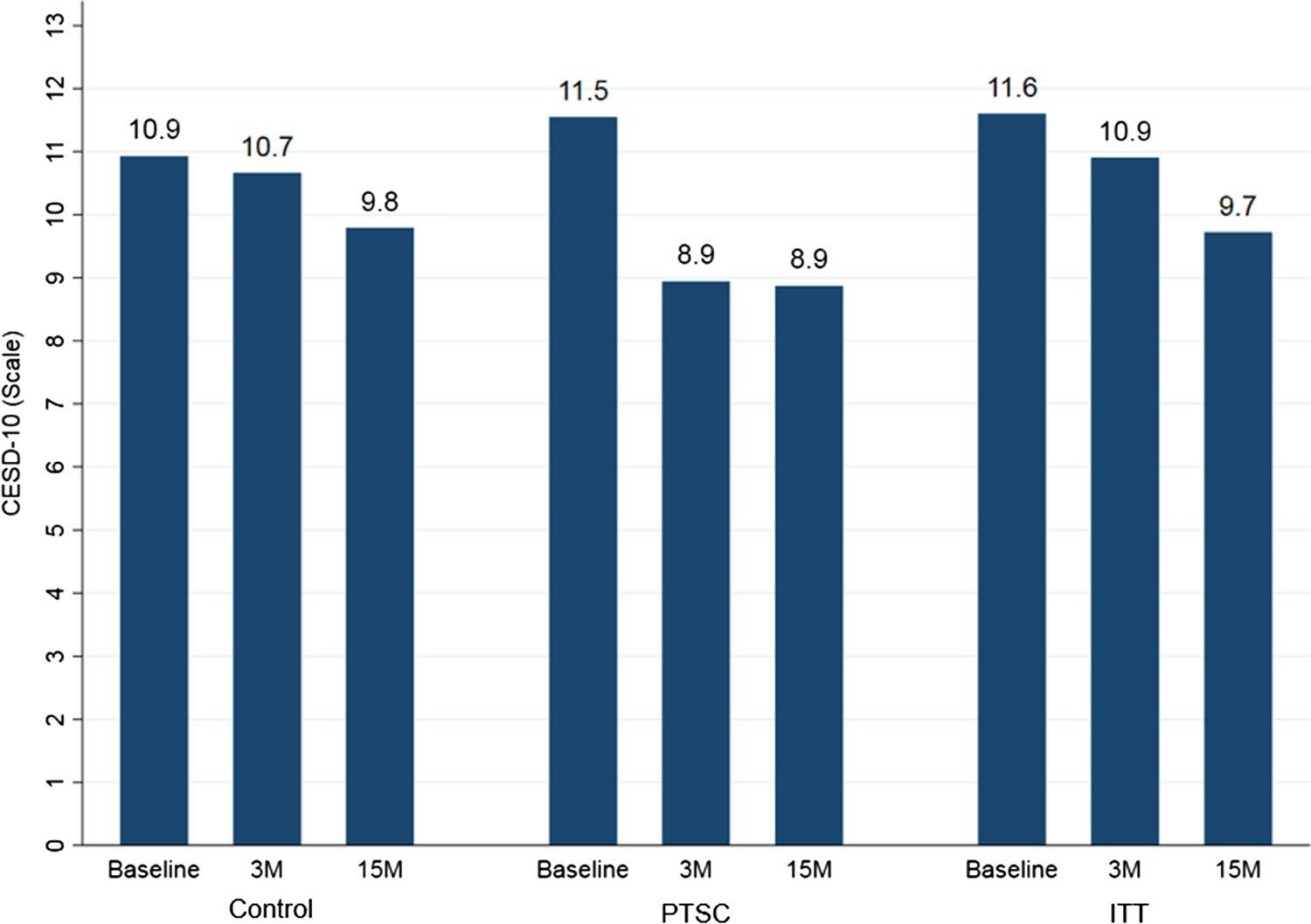
Comparing CES-D-R score in AA women with uncontrolled hypertension in a balanced sample among control, PTSC, and ITT groups.

Figure 2 compares CES-D-10 scale scores between control, PTSC, and ITT groups across baseline, three months, and 15 months surveys. In the PTSC group, the CES-D-10 score significantly declined by 2.6 points, from 11.54 points (95% CI: 10.36–12.73) at baseline to 8.94 points (95% CI: 7.77–10.11) at 3 months (22.5% reduction, *p* = 0.002). It also reduced by 2.7 points from baseline to 8.87 (95% CI: 7.50–10.24) at the 15 months mark (23.1% reduction, *p* = 0.004). In the ITT group, the CES-D-10 score reduced slightly from baseline (11.60 points; CI: 7.71–15.49) to 3 months (10.91 points; CI: 8.62–13.19), but this 5.9% reduction was not statistically significant (*p* = 0.737). Self-reported depressive symptoms in the ITT group rose slightly from baseline to 15 months (13.4% increase, *p* = 0.351), but this was insignificant. Finally, in the control group, the CES-D-10 score did not change substantially from 10.93 points at baseline (95% CI: 9.49–12.37) to 10.66 points at three months (2.5% reduction, *p* = 0.783), and was not significantly reduced at 15 months (9.79 points, 10.4% reduction, *p* = 0.247).

Table 2 displays the results of the fixed effects models ascertaining differences between the control and PTSC in the CES-D-10 scores over time. The parameter of interest in these models is the interaction between the group membership (PTSC vs. control) and the measurement timing (baseline vs. three months or baseline vs. 15 months). Panel A columns 1 and 2 show that, compared to women in the control group, women who participated in the 13-week intervention program had a significantly greater reduction in their CES-D-10 scores from baseline to 3-month follow up (Coeff.: -1.99, 95% CI: -3.57, -0.41) in the unadjusted model. Results remained similar in the adjusted model (Coeff.: -1.99, 95% CI: -3.49, -0.49), and we found similar results at 15 months. Those who participated in the PTSC intervention had a significantly greater reduction in the CES-D-10 score from baseline to 15 months follow up (Coeff.: -2.42, 95% CI: -4.04, -0.80) in the unadjusted model and (Coeff.: -2.68, 95% CI: -4.51, -0.84) in the adjusted model (See Columns 3 and 4). Table 2 also contains the results of the regression models used to conduct per-protocol analyses in Panel B. The results of the adjusted model indicate that women who participated in the 13-week PTSC intervention had significantly reduced CES-D-10 scores from baseline to 3-month follow-up (-2.14; 95% CI: -3.98, -0.30) and baseline to 15 months follow up (-2.22; 95% CI: -3.98, -0.38).

**Table 2:** Regression Model Estimates on the Impact of PTSC on CESD -10 Score

	Baseline vs. three months				Baseline vs. 15 months			
	Unadjusted (Column 1)		Adjusted (Column 2)		Unadjusted (Column 3)		Adjusted (Column 4)	
	Coeff	95% CI	Coeff	95% CI	Coeff	95% CI	Coeff	95% CI
Panel A: Intent-to-Treat analysis								
PTSC status (Ref. Control group)								
PTSC	0.62	[-1.12, 2.37]	0.60	[-1.09, 2.29]	0.62	[-1.16, 2.41]	0.56	[-1.18, 2.31]
PTSC Interaction with 3 M/15 M	-1.99*	[-3.57, -0.41]	-1.99**	[-3.49, -0.49]	-2.42**	[-4.04, -0.80]	-2.38**	[-3.94, -0.83]
Observations	273		273		273		273	
Panel B: Per-Protocol Analysis								
PTSC	0.16	[-1.67, 1.98]	0.31	[-1.46, 2.07]	0.16	[-1.76, 2.07]	0.29	[-1.59, 2.17]
PTSC Interaction with 3 M/15 M	-2.14*	[-3.98, -0.30]	-2.11*	[-3.89, -0.38]	-2.22*	[-4.14, -0.29]	-2.17*	[-4.03, -0.31]
Observations	209		209		209		209	

^*^p < 0.05, **p < 0.01, ***p < 0.001 CI: Confidence Interval

## Discussion

In this study, we used the CES-D-R (CES-D-10) scale to measure self-reported depressive symptoms among low-income AA women between 40 to 75 years old with uncontrolled hypertension who participated in PTSC program in Washington, DC between 2017–2018. Our findings showed that the PTSC intervention effectively reduced women's depressive symptoms at 3 months and 15 months after the intervention. To our knowledge, there are very few studies to which we can compare our results. However, the decrease in depressive symptomology is similar to other group-based interventions. The reductions we observed may be due to aspects of the Sister Circles themselves, insofar as PTSC provided an environment that helped participants gain stress management skills with their peers. Being in a group of women with similar chronic diseases and mental health issues helped participants discuss their own situations, which could have lowered their stress and ameliorated other aspects of their psychosocial health associated with depression.^39–41^

Findings from our previous work showed that low-income AA women with hypertension have unmet depression care needs. Our cross-sectional analysis revealed that a majority of the women in the PTSC-RCT had experienced some level of depression, measured by CES-D-10 scores greater than 10.^14^ Based on the results of our current analysis, about 60% of participants with depression symptoms reported receiving some form of mental health services within the past six months, and about the same amount (58%) reported seeing a mental health professional. We expect these mental health needs to have increased as the COVID-19 pandemic persists and will continue after the SARS-COV-2 virus becomes endemic.^42^ Since the pandemic disproportionately impacts low-income and AA communities, we anticipate that depression care needs will remain high among low-income AA women. This affirms the need for public health and healthcare policymakers, practitioners, and advocates to implement policies and programs that address the unmet depression care needs of this population. Given the high depressive symptoms among low-income AA women with hypertension, we recommend promoting awareness and strategies to diagnose people with depression as early as possible. One approach could include incorporating an assessment of depression symptoms within routine bi-annual screening for low-income AA women with hypertension. Our study also reinforces the critical importance of developing, implementing, and evaluating community-based interventions that are contextualized to low-income AA women’s needs. We found strong evidence to suggest that a culturally responsive intervention, such as the PTSC Program, can reduce depressive symptoms among low-income AA women with hypertension receiving care at a facility.

There is an overall lack of access to treatment for mental illness in Americans, particularly among Black/African Americans.^43^ Several barriers have been enumerated by the American Psychiatric Association (APA) and other bodies and include, but are not limited to: (1) lack of culturally adopted mental health treatments, (2) lack of access to high-quality care, (3) stigma and negative attitudes and beliefs ascribed to mental health conditions, (4) lower rates of mental health providers with diverse racial backgrounds, and (5) inability to pay for treatment because of lack of income or a lower rate of health insurance.^43–45^ The PTSC partially addresses these barriers by offering a culturally tailored, community-based intervention that uses a peer group format to combat stigma and negative attitudes and trained lay facilitators to extend and support mental health providers. Public and private insurance should consider providing reimbursement to support interventions such as the PTSC.

This study has several important limitations. First, the study population was AA women ages 40–75 with low-income and uncontrolled hypertension who received primary care from a facility in Washington, DC. Our findings may not be generalizable to higher-income AA women or low-income AA women who cannot maintain consistent access to care. Second, the primary outcome of this RCT was hypertension, and the CES-D-10 scale (which is a screening tool) was collected as the secondary outcome to measure potential changes in depressive symptoms. While we powered the PTSC-RCT to detect changes in hypertension outcomes, we did not conduct power analyses to discern the impact of the intervention on depressive symptomology. This limits our ability to conclusively state that the PTSC intervention was solely responsible for reducing self-reported depressive symptoms among our study population. Third, we assumed the randomness of missing data with respect to our analyses; yet, given the sociodemographic profile of our study population, it is likely that missingness was linked to adverse social determinants of health that precluded full participation in the intervention and data collection strategies. Finally, we did not include other measures of psychosocial distress in the analyses, partially due to the unavailability of data.

Despite these limitations, this study has several strengths. The PTSC-RCT measured depressive symptoms in a largely homogeneous population with respect to race/ethnicity, socioeconomic and demographic characteristics, health status, and geography^.^ Further, because participants in this study had received their care at a facility, it is reasonable to consider that undiagnosed depression was not due to a lack of access to care. Indeed, the association between depressive symptoms and hypertension underscores the need for integrated models of care that facilitate access to mental health support for women with high blood pressure. It also reinforces the importance of strategies to monitor both the quality of mental health services provided and the extent of the uptake of such services to ensure receipt of adequate treatment.^46^ Since the CES-D-10 is widely used to screen depressive symptoms, our study results are comparable to national survey results. Finally, there is a strong need to develop, implement, evaluate, and disseminate promising community-based interventions that reduce depression symptoms among AA women. This study builds our knowledge of effective interventions and can help equip lower-income Black women with much-needed tools to recognize symptoms of depression and seek help.^47^

## Conclusion

The prevalence of depression is high among women with chronic diseases, especially hypertension, specifically African American women with lower socioeconomic status. Addressing the depression care needs of these women is a multidimensional mission that requires cooperation among mental health specialists, primary care providers, community-based programs, researchers, and public health officials. Interventions should consider addressing social determinants of health, including individual, sociocultural, environmental, and historical factors that impact the mental health of low-income African American women. There is a need for programs such as PTSC that empower women and help them better manage their emotional and physical health issues and depressive symptoms. Our findings show that free, culturally competent, community-based intervention programs such as the PTSC can effectively reduce depressive symptoms.
